# Innovation facilitators and sustainable development: a country comparative approach

**DOI:** 10.1007/s10668-023-03055-w

**Published:** 2023-03-05

**Authors:** Nuria Chaparro-Banegas, Ana Maria Ibañez Escribano, Alicia Mas-Tur, Norat Roig-Tierno

**Affiliations:** 1 Department of Economics and Social Sciences, Universitat Politècnica de València, Camino de Vera S/N, 46022 Valencia, Spain; 2 Department of Corporate Finance, Universitat de València, Tarongers, S/N, 46022 Valencia, Spain; 3 Department of Management, Universitat de València, Tarongers, S/N, 46022 Valencia, Spain

**Keywords:** SDGs, Cluster analysis, Innovation, Economic growth, Sustainable development, Innovation facilitator

## Abstract

National and international organizations have introduced policies aimed at sustainable development. These policies are designed to encourage sustainable forms of business to meet the Sustainable Development Goals (SDGs) of the 2030 Agenda. Regional inequalities in sustainable development may be exacerbated by disparate levels of innovation. This paper analyzes the variations between clusters of countries according to the degree to which they have achieved the SDGs and their levels of innovation facilitators. Two types of analyses were employed. First, cluster analysis was used to examine changes in groups of regions with similar innovation characteristics between 2015 and 2020. Data for 122 countries were gathered from the World Bank, the SDG Index, and the Global Innovation Index. Second, multiple linear regression analysis was used to assess the power of the variables in the model to explain the level of sustainable development. The results reveal four clusters (low, medium, high, and very high innovative facilitators and sustainable development), as well as movements between those clusters from 2015 to 2020. The multiple linear regression analysis shows that the variables have explanatory power with respect to the dependent variable of sustainable development. This analysis also reveals different degrees of importance of the variables for each cluster. The findings highlight the need to consider the limitations of economic growth in terms of innovation facilitators to promote sustainable development. If policymakers recognize the limitations of economic growth and the physical ecosystem, degradation of the environment can be avoided, even when there is innovation. Global and individual social welfare can thus be ensured. This study offers valuable insights into how to achieve sustainable development through innovation facilitators by providing in-depth knowledge of the individual characteristics of innovation systems and considering the limitations of economic growth.

## Introduction

For decades, international organizations and governments have recognized the challenges facing different regions around the world. According to the United Nations (2021c), these challenges are not only economic (e.g., driving economic growth, stimulating investment, and reducing unemployment) but also social and environmental (e.g., poverty and climate change). Failure to tackle these challenges could have negative consequences for society by limiting its ability to meet its own needs. These global problems have been exacerbated by the COVID-19 pandemic, which has highlighted the importance of employment, income, and social protection systems (United Nations, 2021a), among other issues.

Given the global situation in light of these challenges and the growing concern of governments, sustainable development policies have been introduced. The World Commission on Environment and Development brought the concept of sustainable development to the forefront of the political agenda, defining it as “meeting the needs of the present without compromising the ability of future generations to meet their own needs” (Brundtland, 1987; p. 24). This definition integrates the three dimensions of sustainability (i.e., the economy, society, and the environment). However, many scholars disagree with this institutional view (e.g., Daly, 1974; Kallis et al., 2018), arguing that the economic dimension of sustainability should be kept in check because economic growth can trigger environmental degradation and destruction (Fournier, 2008). These arguments are aligned with the post-growth literature, which suggests that the contribution of economic growth to sustainable development is limited, placing emphasis on the role of the social and environmental dimensions of sustainability.

Not only has the United Nations (UN) highlighted the importance of sustainable development through its eight Millennium Development Goals (MDGs) and its subsequent 17 Sustainable Development Goals (SDGs), but many countries have also included sustainability measures in their political strategies. For example, the European Union (EU) has the next-generation EU, European Green Deal, and Horizon Europe. The participation of private companies (United Nations, 2015a), universities, and research institutions (Horbach, 2016) in sustainable development is also essential because they contribute through research and development (R&D), project financing, job creation, and trade.

The United Nations Development Program has adopted innovation, technology (Omri, 2020), and entrepreneurship (Filser et al., 2019) as fundamental pillars of the three dimensions of sustainable development. First, innovation and knowledge can contribute to improving people’s living conditions in areas such as transport, production, medicine, and energy (Szopik-Depczyńska et al., 2018a). Second, all sustainability dimensions can be positively influenced by the outcomes of new technologies (de Queiroz Machado et al., 2021). In addition, entrepreneurship in all its forms has become one of the main economic and social drivers of innovation (López-Rubio et al., 2020, 2021). For these reasons, it is important to identify the interactions between economic, institutional, knowledge or human, and environmental systems (Szopik-Depczyńska et al., 2018b). This paper refers to these systems as innovation facilitators influencing both innovation activities and the achievement of sustainable development. However, innovation and technological progress could negatively affect society because innovation and technology imply an increase in pollution and material and energy use (Fournier, 2008). Moreover, innovation and technology could also foster inequalities between rich and poor countries (Raffer & Singer, 2002), including among individuals in terms of, for example, spatial and demographic distribution or abilities or infrastructures (Sovacool et al., 2022). Other adverse effects of innovation relate to digitalization, the Internet, and big data storage, all of which entail vast amounts of carbon emissions, water use, and land footprint (al Kez et al., 2022).

In recent decades, sustainability-oriented innovation has become an intensely debated topic in the literature because both concepts (i.e., sustainability and innovation) involve social and ecological aspects in organizational structures, products, and processes (Klewitz & Hansen, 2014). Different actors participate in these innovations and concerns, which are regarded as drivers of sustainable development (Mulgan, 2006). Nevertheless, there are regional differences in the ability to promote innovation systems. These differences have hindered real opportunities for sustainable development across countries (Omri, 2020).

Innovations can exacerbate the inequalities between countries, leaving many unable to achieve sustainable development. This argument is consistent with the institutional view of sustainable development, which emphasizes the role of innovation in driving the economic, social, and environmental dimensions of sustainability. Accordingly, other approaches to sustainable development (e.g., from the post-growth literature) are ignored. Therefore, the question is, are innovation facilitators (including economic growth) positively influencing sustainable development? Also, what is their real impact? In answer to these questions, this paper has two aims. First, it analyzes variations between country clusters based on the degree to which they have achieved the SDGs and their innovation facilitators (a country’s economic, institutional, knowledge, and environmental facilitators) at different times. That is, the study answers the question of why countries that had similar characteristics in 2015 had shifted into other clusters by 2020. The movements between clusters over that period are captured and identified, as are the causes of different levels of sustainable development and innovation facilitators. Therefore, the differences in the sustainable development of regions are also identified. Cluster analysis was conducted to (1) group economies with similar innovation levels and characteristics and (2) study changes in these clusters of economies between 2015 and 2020. Second, this paper assesses the extent to which the variables in the model explain sustainable development, both at the overall sample level and at the individual cluster level. Two multiple regression analyses were conducted: one for the overall sample and another by clusters. The multiple regression analysis by clusters shows whether the explanatory power of the independent variables changes by cluster, with each cluster characterized by a different level of sustainable development.

Given the innovation drivers of economies, this paper presents a model based on five innovation facilitators provided by the World Bank, the SDG Index, and the Global Innovation Index (GII). These facilitators are considered important for capturing the economic, institutional, knowledge (or human), and environmental dimensions that could influence sustainable development (Rosca et al., 2018; Szopik-Depczyńska et al., 2018b; Yuan & Zhang, 2020). The five selected facilitators are GDP per capita, SDG Index, institutional framework, human capital and research, and ecological sustainability. The institutional framework, human capital and research, and ecological sustainability are considered facilitators of innovative activities within a country (Cornell University et al., 2020). The analysis in this study is based on detailed data for 122 countries. The results reveal four clusters of countries (low, medium, high, and very high innovative facilitators and sustainable development) with similarities in terms of these innovation facilitators. The results also reveal movements between clusters from 2015 to 2020. The originality and novelty of this study lie in the crucial insights that emerge from the combination of the temporal analysis of country movements and the multiple regression analyses. This dual analysis (1) shows the existence of a link between innovation and sustainable development through innovation facilitators and (2) reveals that the influence of economic growth on sustainable development is limited. Therefore, strategies that lead to unconstrained economic growth can result in environmental degradation and, ultimately, the failure to achieve sustainable development.

This paper is organized into several sections. The second section of this paper describes the framework for sustainable development, taken as a global reference, and other perspectives on sustainable development. The third section presents the factors in the model and describes the relationship with sustainable development specified in the literature. The fourth section describes the method and indices used in the analysis. The results are explained in the fifth section. Finally, the last section concludes and presents the limitations of the study and future research possibilities.

## Theoretical framework

### The SDGs as a sustainable development agenda

Sustainable development involves complexity and uncertainty for all countries and regions. Therefore, policymakers and other economic actors must identify new tools to assess the risks that may arise from decision making. Measures and actions to protect sustainability can then be introduced (Firoiu et al., 2019). In 2000, the UN created eight Millennium Development Goals (MDGs) to reduce extreme poverty, provide universal primary education, and halt the spread of HIV/AIDS. This plan was ratified by international organizations and countries around the world, which pooled their efforts and resources to satisfy the needs of the poorest and most vulnerable (United Nations, 2021d). However, in 2015, the failure to achieve these goals led to the creation and adoption of the 2030 Agenda for Sustainable Development (United Nations, 2021b).

Despite its multifaceted and complex character, the 2030 Agenda has become a reference framework for many nations by upholding human rights, humanity, and nature (Firoiu et al., 2019). This action plan introduced 17 goals separated into 169 targets covering the economic, social, and environmental dimensions of sustainable development. Initial progress in tackling the SDGs has been gradual because the idiosyncrasies of each country or region make this task difficult. For this reason, communication and information about SDGs and sustainability have become crucial to stimulate the commitment, participation, and interest of individuals worldwide (Firoiu et al., 2019).

The alarming situation in the face of global challenges (e.g., the health crisis caused by COVID-19, climate change, protection of human rights, and international law and justice) shows the need to create a global alliance to replace the current alliance, which has failed to achieve clear outcomes. Such a global alliance of both developed and developing countries would help eradicate poverty, improve education and health, boost sustainable economic growth, reduce inequality, address climate change, and conserve ecosystems (United Nations, 2021b). The 17 SDGs were created not only to tackle these challenges but also to address the implementation of actions designed to do so, as well as providing a framework for review and follow-up.

The 2030 Agenda places the focus on people, planet, and prosperity by encouraging global society to act against situations of injustice. The SDGs are interrelated, which is a crucial part of improving the quality of life and well-being of people around the world (United Nations, 2015b). Five dimensions are addressed by the SDGs. The *People* dimension mainly relates to health, education, gender, and poverty. *Planet* refers to the importance of respecting the environment at all levels. *Prosperity* refers to ensuring global economic, social, and environmental prosperity. *Peace* relates to fostering peaceful, inclusive, and just societies. *Partnership* refers to achieving a global alliance committed to sustainable development. However, the reality of the 2030 Agenda and the SDGs means that countries may face numerous tradeoffs to achieve sustainable development (le Blanc, 2015).

### Sustainable development: other approaches

In the literature, there is no consensus on the definition of sustainable development, which leads to different interpretations and responses (Mebratu, 1998). Although the institutional view of the UN and other international organizations reconciles economic growth with the resolution of social and environmental problems, many authors disagree (e.g., Daly, 1974; Kallis et al., 2018). The UN’s conceptualization lacks theoretical development (Purvis et al., 2019), assuming that economic growth is required to reduce poverty and environmental degradation through more accessible markets (Castro, 2004). However, this type of growth causes environmental destruction (Fournier, 2008).

Hopwood et al. (2005) argued that economic growth implies a progressive increase in the use of resources, which, in turn, generates an increase in waste production. Production negatively impacts the environment (Giljum et al., 2005). Therefore, an unsustainable situation arises, preventing sustainable development. The post-growth literature considers the limits of growth, explaining that resources are finite and that the population cannot grow indefinitely. Any economic effect on the ecosystem generates a physical transformation (Daly, 2018), which has consequences at all levels of society. Given the diversity of perspectives and approaches to sustainable development, this section presents some of the arguments from the post-growth literature, contrasting with the sustainable development institutional view and revealing arguments that illustrate the limitations of economic growth.

One of the key concepts in the post-growth literature is degrowth. Degrowth is known as “a process of political and social transformation that reduces a society’s throughput while improving the quality of life” (Kallis et al., 2018, p. 292). Degrowth is based on the proposition that human development without economic growth is possible (Schneider et al., 2010). Similarly, the concept of a steady-state economy is also found in the post-growth literature. A steady-state economy refers to the existence of a stable population and wealth, maintained at a desirable level and determined by a low level of production (Boulding, 1970; Daly, 1974). The steady-state economy approach is based on the fact that people establish goals by considering the preservation of the physical ecosystem and its limits (Daly, 2018).

Daly (1990) distinguishes between growth and development, identifying the qualitative development of non-growing countries and systems over long periods. The global ecosystem is finite. It does not grow but does develop. Since the economy is one of the areas within this global ecosystem, it is impossible to drive economic growth indefinitely or for long periods. The author claims that growth pushes the economy beyond the optimal point of physical dimensions, damaging the biosphere and increasing poverty. These arguments suggest that economic growth is an unsustainable goal that negatively affects society and the environment, undermining the opportunities of the present and future generations by exceeding nature’s limitations.

Sustainable development involves moving away from the growth economy and moving toward a steady-state economy that includes both the Northern and Southern Hemispheres (Daly, 1996). Throughout history, there has been a distribution of wealth between the Northern and Southern Hemispheres. The existing Northern–Southern Hemisphere relationship is not sustainable because of their economic interdependence. Economic interdependence is the continued dependence of the Southern Hemisphere countries on the influential countries of the Northern Hemisphere in terms of resources, trade, information and knowledge flows, and other aspects. However, the economic interdependence of Northern Hemisphere countries relies on the opportunity to exploit any type of resource, such as natural or human capital resources (Shariff, 1997). Therefore, there is a need to foster a new relationship between rich and poor countries from the Northern and Southern Hemispheres in which they partner with each other (Raffer & Singer, 2002).

According to several authors in the post-growth literature (e.g., Schneider, 2003; Schneider et al., 2010), research, innovation, and technology should be oriented toward lower consumption through policy, technological, and lifestyle instruments. These instruments should impose material and energy use limits while continuing to encourage consumption. Despite the design and implementation of eco-efficient innovations, they still encourage consumption and production, leading to higher energy use, pollution, carbon emissions, and other negative effects (Fournier, 2008). This idea is aligned with the concept of rebound effects, which explain the non-decrease in energy consumption despite continuous improvement in technical energy efficiency. Lange and Berner (2022) showed that, through several rebound instruments, energy efficiency improvements trigger economic growth, thus raising energy demand. Many countries and international organizations have promoted the transition to renewable energy (Sovacool & Dworkin, 2012) because of the perceived benefits to society (Liang et al., 2019). However, energy transitions create injustices at the local, national, and global levels (Sovacool et al., 2019). These energy injustices are linked to the environment, community health, energy prices, unequal access to energy, circulation of waste, and other areas. Therefore, although eco-efficient technologies and innovations are valuable, they negatively affect the environment by triggering excessive consumption and use of natural resources that exceed biophysical limits.

Another key concept in the post-growth literature is absolute decoupling. According to the institutional view, which reconciles economic growth with social and environmental development, promoting absolute decoupling is necessary to achieve sustainable development. Many policymakers aim to achieve absolute decoupling (Giljum et al., 2005) because they believe that it is the means to achieve sustainable development. Absolute decoupling implies an absolute reduction in environmental pressures while economic growth accelerates (Giljum et al., 2005). The result will be that the resource efficiency rate (GDP/resource use ratio) exceeds the increase in GDP. However, cases of absolute decoupling are rare. They are related to low economic growth and the increase in imports of material-intensive goods (Otero et al., 2020). They also occur over short periods.

Other theories claim that technology and knowledge are joined by a process of feedback. Countries with a higher and more complex understanding of innovation facilitators have more opportunities to improve national innovation systems. A vicious circle arises in which the poorest countries tend to continue to have high levels of poverty, high social exclusion, and low levels of growth. To reduce the gap between the Northern and Southern Hemispheres, innovation facilitators in poor countries need to be stronger and more effective (Raffer & Singer, 2002). However, innovation facilitator and innovation system characteristics of developing countries remain unexplored in the literature (Choi & Zo, 2019; Khan, 2022). Low- and medium-income countries absorb knowledge from abroad to create value for their local communities (Khan, 2022). Fernández et al. (2021) showed differences in the innovation facilitators of developed and developing countries. Developing countries rely more on collaboration, alliances, and networks. They acquire software, equipment, or machinery. They use external R&D and innovation and knowledge sources. Public support and market factors play a secondary role.

#### Research model: selected innovation facilitators and their link to sustainable development

**Fig. 1:**
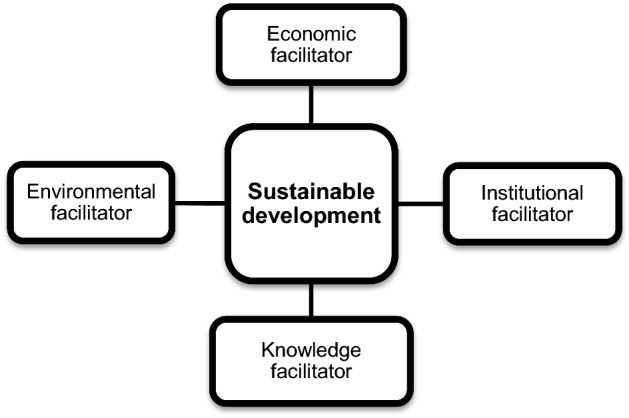
Proposed research model

The literature identifies numerous drivers of national development such as wealth, health, education (Todaro & Smith, 2020), technological development, a country’s fiscal situation, and investment (Soliyev & Ganiev, 2021). In this paper, five innovation facilitators (dimensions) form the basis of the research model shown in Fig. 1. These facilitators are sustainable development, economic, institutional, knowledge, and environmental, proxied using the SDG Index, GDP per capita, institutional framework, human capital and research, and ecological sustainability, respectively. The variables institutional framework, human capital and research, and ecological sustainability are considered facilitators of innovative activities within a country (Cornell University et al., 2020). The aim is to use these variables to create groups of countries with similar national characteristics in terms of their sustainable development.

Some governments have voluntarily created indicators to review their achievement of the SDGs at the national level (Schmidt-Traub et al., 2017). However, these indicators lack international harmonization and thus comparability. The *SDG Index* is a global instrument that provides detailed information on sustainability (Kroll, 2015). This index can be used to assess and compare individual countries. The index also enables the measurement of countries’ degree of achievement of the SDGs (Sachs et al., 2020), providing a set of indicators that are easily understandable and accessible, as well as comprehensive. Inclusion of this variable in the model is justified by countries’ growing concern in achieving sustainable development. This variable provides information on the multifaceted characteristics of each country, reflecting the situation of each country in terms of sustainability (Kroll, 2015). However, although this paper uses the international institutional view of sustainable development (based on the three dimensions of sustainability), the post-growth literature suggests that unlimited economic growth hinders the achievement of sustainable development. In other words, economic growth stops contributing to human welfare when a certain economic level is reached due to the rising environmental degradation.

Under this institutional view, *economic facilitators* (represented by GDP per capita) would also contribute to the achievement of sustainable development. Spaiser et al. (2017) showed that wealth generally increases socioeconomic inclusion and reduces poverty. These results are in line with the findings of Hamilton and Hepburn (2014), who found a close link between wealth and sustainability, implying a decrease in future well-being when the real wealth of an economy declines. Some authors have argued that accelerating economic growth can trigger faster integration of innovation in different areas of society (e.g., Bircan & Gençler, 2015). The Kuznets curve hypothesis (Kuznets, 1955) states that economic growth leads to an increase in income inequality until a certain level of national or regional income is reached. After that income level, inequality begins to decrease. In addition to reaching this level of income, the country or region must have developed institutionally, industrially, and in terms of welfare. Therefore, according to the Kuznets curve, the relationship between economic growth and income inequality follows an inverted U-shaped curve.

Nadeem et al. (2020) found that both short- and long-term innovation is positively influenced by wealth. Innovation is based on the combination of new knowledge and existing knowledge (Awan et al., 2019), which, through a better understanding of business behaviors and processes, could promote sustainable innovation (Grabara et al., 2020). Therefore, the literature suggests that a country’s level of wealth, as an innovation facilitator, also contributes indirectly to sustainable development.

**Hypothesis 1:** *A country’s wealth positively contributes to sustainable development until a certain level of economic progress.*

The *institutional facilitators* (under the institutional framework) are defined as the formal (laws, rights, constitutions, etc.) and informal (customs, sanctions, traditions, codes of conduct, etc.) norms that constrain (Periac et al., 2018), stimulate, or stabilize economic, political, and social relations. Often, institutional structures encourage the achievement of welfare goals by helping establish measures related to health, gender equality, and education (Waage et al., 2015). Moreover, an improvement in the institutional framework (e.g., political stability and control of corruption) raises environmental quality levels (Khan et al., 2022). The attainment of sustainable development still largely depends on the participation of citizens (Leal Filho et al., 2018). The implication is that if the population is unaware and uninterested in sustainable activities and innovations, the country will be unable to progress in its sustainable development. In addition, companies include more SDGs in their sustainability reports when their home countries have certain institutional and organizational features such as employment protection policies or a national corporate social responsibility strategy (Tsalis et al., 2020). In some cases, institutions do not have a clear, holistic view of the determinants of sustainable development or of sustainable development itself (Sedlacek & Gaube, 2010). However, a society’s lack of interest, awareness, and institutional knowledge may not be the only reason institutional facilitators fail to stimulate the design and implementation of measures to promote sustainable development. This view would be narrow and biased. The responsibility for sustainable development lies not only with citizens but also with all actors in society, including governments, private institutions, companies, universities, and research institutions. For example, Howes et al. (2017) argued that policy failure in driving sustainable development arises for several reasons, including policy conflicts of interest, inadequate administrative resources and implementation of incentives, and lack of policy objective specifications.

An institutional structure that supports sustainable practices can help achieve sustainable development by encouraging changes in technology R&D, marketing models, or financial structures (Yuan & Zhang, 2020). Moreover, institutions and other organizations also play a supporting role in the innovation process (López-Rubio et al., 2020). Therefore, the institutional facilitators are also essential to promote innovations (Cornell University et al., 2020) that encourage national and regional sustainable development. The implication is that the government and the institutional structures play a key role in encouraging the creation of sustainable development and innovation.

***Hypothesis 2:*** *A country’s institutional facilitators have a positive effect on the achievement of sustainable development.*

The literature shows that *knowledge facilitators* such as human capital and research influence sustainable development. Human capital is a society’s set of knowledge, skills, motivations, and competencies capable of generating social well-being (Chikwe et al., 2015). Research involves studying properties and characteristics of concepts to discover information (Abali et al., 2019; Okeke, 2004). Throughout the twentieth century, economic theories about aspects that promote global economic growth, such as education, R&D, and patents, have emerged (Pelinescu, 2015). To drive sustainable development, human capital can influence environmental quality because of the link between education, environmentally friendly behavior, and environmental awareness (Ahmed et al., 2020; Chankrajang & Muttarak, 2017).

The development of human capital through higher education stimulates not only socioeconomic development but also innovation at the national, regional, and local levels (Garcia-Alvarez-Coque et al., 2021). Higher education institutions are able to engage diverse stakeholders in learning activities based on sustainability (Baumber, 2021). Çakar et al. (2021) found that human capital can decrease environmental degradation while boosting economic growth. This finding indicates that human capital development leads to sustainable development by boosting the national economy while reducing pollution, which positively impacts society’s welfare. Similarly, Diaconu and Popescu (2016) reported that human capital is a key element in sustainable development because it drives the three dimensions of sustainability. First, it drives economic sustainability because the greater productivity and creativity of healthier and more educated people boosts economic growth. Second, it drives social sustainability because greater development of human capital guarantees social satisfaction and therefore fosters cooperation and social well-being. Third, it drives environmental sustainability as a result of increased awareness of environmental issues.

**Hypothesis 3:** *Knowledge facilitators encourage sustainable development.*

Finally, *environmental facilitators* (represented by ecological sustainability) can also influence the achievement of sustainable development. Ecological sustainability is the long-term ability to continue living given the limitations of the biophysical world (Porritt, 2007). Ecological development based on ecological sustainability aims to minimize environmental pollution and resource exploitation by reducing the production and use of harmful substances (Littig & Grießler, 2005). From an ecological sustainability perspective, unlimited economic growth is impossible. Therefore, sustainable development has the potential to improve means and ends by recognizing the limitations of nature (Borland et al., 2016; Ekins, 2000). In contrast, other authors (e.g., Tomislav, 2018) claim that ecological sustainability is a fundamental element in driving economic and sociocultural sustainability. Nevertheless, it continues to be a topic of discussion within the framework of sustainable development. This discussion suggests that ecological sustainability may affect other dimensions. Therefore, focusing more resources or efforts on ecological sustainability could drive sustainable development more rapidly.

**Hypothesis 4:** *Environmental facilitators foster the attainment of sustainable development.*

Table 1 summarizes the variables in the model.

**Table 1:** Description of innovation facilitators (dimensions) included in the model

Innovation facilitator	Variable	Definition	Source
Sustainable development	SDG Index	Performance of countries in achieving the SDGs	SDG Index Report
Economy	GDP per capita	Sum of goods and services produced in a country over a given period divided by average population (OECD, 2014)	World Bank Data
Institutions	Institutional framework	Formal and informal rules or norms that structure economic, political, and social interaction	Global Innovation Index
Knowledge	Human capital and research	Set of knowledge, skills, motivations, and competencies of society that can be enhanced through research (study of the characteristics of concepts)	Global Innovation Index
Environment	Ecological sustainability	The ability to ensure the long-term survival of future generations by minimizing environmental pollution and resource exploitation, as well as considering the limitations of nature	Global Innovation Index

Source: Authors based on Okeke (2004), Littig and Grießler (2005), Porritt (2007), OECD (2014), Chikwe et al. (2015), Kroll (2015), Periac et al. (2018), Abali et al. (2019), Sachs et al. (2020), and Sautet (2020)

## Data and method

### Data

The study used data from the SDG Index, Global Innovation Index (GII), and World Bank. Data on 122 countries were gathered for the years 2015 and 2020. The year 2015 was selected because it was when the 17 SDGs were adopted, while 2020 was the latest year with available data. The World Bank offers a data analysis and visualization tool for various topics such as health, corruption, economic growth, and poverty. The tool uses time-series data. It is flexible, allowing the creation of tables and graphs that are easy to share and save (World Bank, 2021). The GDP per capita values (economic facilitator) were gathered from the World Bank database. The GDP per capita data for the year 2019 were used because those for the year 2020 were not yet available at the time of the study.

The SDG Index and Dashboards provide a set of indicators for monitoring attainment of the SDGs and for complementing the standardization and compilation by national and international organizations (SDG Index, 2021). The data on the SDG Index (sustainable development) were collected from the SDG Index and Dashboards database. In the case of this variable, data for 2016 were collected because data for 2015 were not available. This data unavailability is one of the limitations of the study.

The GII captures the characteristics and trends of the global and national innovation ecosystems through new approaches and metrics (WIPO, 2021). This index provides data that enable both the assessment of innovative performance and the introduction of new policy measures of innovation. This index provides data on the institutional framework, human capital and research, and ecological sustainability (institutional, knowledge, and environmental facilitators, respectively).

### Method

#### Multiple linear regression analysis

Multiple linear regression (MLR) enables the modeling and examination of a linear relationship between explanatory variables and an explained variable (Field et al., 2012). The aim of the MLR analysis in this study was to identify how accurately the selected independent variables (economic, institutional, knowledge, and environmental facilitators) explain the dependent variable (sustainable development) for the years 2015 and 2020. Stepwise linear regression was used to select or eliminate independent variables because all of these independent variables were considered to have an equal probability of explaining sustainable development. Sustainable development was represented by an indicator (SDG Index) that determines the extent to which countries achieve the SDGs (Sachs et al., 2020). Stepwise linear regression highlighted the variables that provided the model that best fit the data without introducing researcher bias.

#### Cluster analysis

Cluster analysis is the classification of similar objects (also referred to as observations or individuals) into groups where both the number of groups and their form are unknown (Kaufman & Rousseeuw, 2009). In the data mining process, clustering is a useful tool to identify groups or patterns in the underlying data (Frades & Matthiesen, 2010). The main objective of this method is to identify clusters of points in a specific space (Edwards & Cavalli-Sforza, 1965). The categorical structure that best fits the sample observations can thus be determined (Anderberg, 2014). Scholars can also fulfill several other objectives using this methodology, such as classifying objects according to an existing set of clusters or testing the existence of some natural classes of individuals or groups (Härdle & Simar, 2019).

Cluster analysis encompasses a variety of mathematical methods for determining which objects are similar or dissimilar within a group. Objects with similar descriptions are mathematically grouped together in a cluster (Romesburg, 2004). To divide the set of observations into different groups with similar properties, two elements must be selected. The first is a proximity measure (also called similarity or distance measure) by which the similarities of characteristics of each pair of individuals are tested. This proximity measure is used to determine the closeness of objects. The closer the objects are to each other, the more homogeneous they are. Hence, they are included in the same cluster. The second is a group creation algorithm through which allocations are made in such a way that the observations in a group are as close as possible, but the differences between groups are large (Härdle & Simar, 2019).

## Results

### Multiple linear regression analysis

MLR analysis was used to test whether the variables in the model explain sustainable development. Table 2 summarizes the results after estimating different models for the years 2015 and 2020 with sustainable development as the explained variable. In 2015, the four models consisted of several parameters. While Model 1 only included a constant, Model 2 included a constant as well as knowledge facilitators. Model 3 included a constant as well as knowledge and environmental facilitators. Finally, Model 4 included a constant as well as knowledge, environmental, and institutional facilitators. Economic facilitators were not significant and were not included in the model (see Table 4, Appendix 1). This finding is aligned with the literature that decouples economic growth from sustainable development. The coefficient of determination (*R*^2^) for Models 2, 3, and 4 was high (*R*^2^ = 0.765, 0.835, 0.842, respectively). These results indicate that the independent variables in each respective model explain 76.5%, 83.5%, and 84.2% of variation in the dependent variable.

**Table 2:** Summary of the model with dependent variable sustainable development for 2015 and 2020

Model	R	R^2^	Adjusted R^2^	RMSE
*2015 sustainable development*				
1	0.000	0.000	0.000	12.591
2	0.874	0.765	0.763	6.135
3	0.914	0.835	0.832	5.160
4	0.917	0.842	0.838	5.071
*2020 sustainable development*				
1	0.000	0.000	0.000	8.626
2	0.801	0.641	0.638	5.187
3	0.856	0.733	0.729	4.494
4	0.862	0.743	0.736	4.430

*RMSE* root-mean-square error

For 2020, Models 1, 2, and 3 were identical to those for 2015 (Model 1 included the constant; Model 2 included the constant and knowledge facilitators; Model 3 included the constant and knowledge and environmental facilitators). The difference between the models for years 2015 and 2020 resided in Model 4, which included economic facilitators (for the year 2020) instead of institutional facilitators (for the year 2015). Institutional facilitators were not significant (see Table 5, Appendix 1). The values of *R*^2^ indicate that the goodness of fit of the models was lower than in the previous analysis. Models 2, 3, and 4 had values of 0.641, 0.733, 0.743, respectively, with the independent variables in each respective model explaining 64.1%, 73.3%, and 74.3% of the variation in the dependent variable. The models were statistically significant for 2015 and 2020 because the *p* value was less than 0.05 (see Table 6, Appendix 1). Thus, the proposed models adequately explain the dependent variable of sustainable development.

All parameters in the models were significant at the 95% level because the *p* value was less than 0.05. Hence, they had explanatory power with respect to the dependent variable (see Table 4 and 5, Appendix 1). Finally, the variance inflation factor (VIF) indicated no collinearity problems between the variables because the values were less than 10, following the criterion of Kleinbaum et al. (1988).

Multiple linear regression analysis by clusters was also conducted to test whether the explanatory power of the independent variables varied depending on the cluster. (The naming of the clusters is described in the following section.) Cluster 1 (low innovative sustainable development) reflects that a minimum level of national wealth is essential in achieving sustainable development because this type of development cannot be promoted if economic and financial resources are unavailable to meet basic needs. Cluster 2 (medium innovative sustainable development) shows that knowledge facilitators such as human capital are fundamental in promoting the achievement of SDGs. This finding indicates that it is more difficult to achieve sustainable development without an educated and well-equipped society that encourages research and activities based on sustainable development alternatives. In 2015, Cluster 3 (high innovative sustainable development) presented human capital as the variable with the highest significant value. In 2020, institutional facilitators were necessary to drive sustainable development, suggesting that national institutional structures are capable of promoting this type of development. Finally, in 2015, Cluster 4 (very high innovative sustainable development) highlighted institutional facilitators as key elements for achieving the SDGs. In 2020, environmental facilitators had gained greater importance.

This analysis shows that, to promote sustainable development, a minimum level of wealth is first necessary to satisfy basic needs. Subsequently, once the country has reached a certain level of economic development, the most relevant elements for sustainable progress are knowledge, institutional, and environmental facilitators, ordered according to their contribution to sustainable development. These findings reflect that the relevance of economic growth is limited to a certain level. Therefore, when a specific economic, knowledge, and institutional development is achieved, it is possible to invest in strategies, activities, and alternatives that preserve the environment to a greater degree.

### Comparative analysis: a cluster analysis of similar groups

This section presents the results of cluster analysis, where countries with similar characteristics for the years 2015 and 2020 were grouped. Variation between clusters in terms of achievement of sustainable development and innovation facilitators is examined in this section. The possible causes of any movements between these clusters are identified. A k-means cluster analysis was performed using a hard partitioning algorithm. This algorithm divides the data set into different clusters, with each object belonging to a single group. Each cluster consists of data observations that show a maximum degree of similarity between one another and a minimum degree of similarity with objects in other groups (James et al., 2013).

**Fig. 2:**
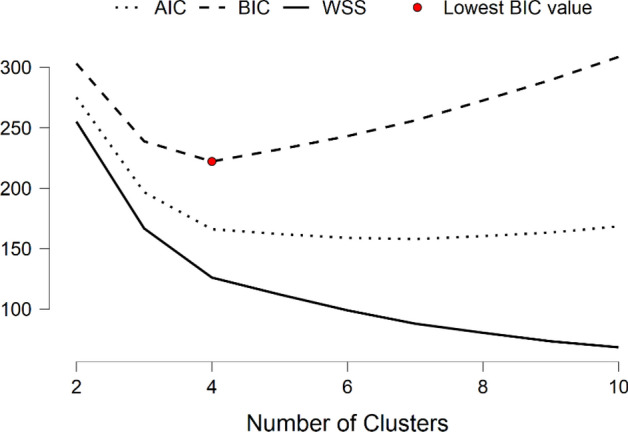
Elbow method plot

Table 3 presents the main data obtained from the k-means cluster analysis for 2015 and 2020. The model was optimized based on the Bayesian information criterion (BIC). The Akaike information criterion (AIC) and BIC are optimization methods to determine the quality of the resulting clusters (James et al., 2013). Both aim to avoid overfitting by penalizing for adding parameters to the model. According to the set of observations of 122 countries, the optimal number of clusters (i.e., the value that minimizes the BIC) was four (Fig. 2). Comparing the value of the BIC for the years 2015 and 2020 shows a slight decrease in the model’s goodness of fit. (Lower scores indicate a better fit of the model.) The value of *R*^2^ was similar for both years (i.e., 79% and 78% in 2015 and 2020, respectively). Thus, the reliability of the model was relatively high.

**Table 3:** 2015 and 2020 k-means clustering

	Clusters	*N*	*R*^2^	AIC	BIC	Silhouette
2015	4	122	0.79	166.07	222.15	0.37
2020	4	122	0.78	174.58	230.66	0.38

The model is optimized with respect to the BIC value

The level of similarity of the objects with other objects in the same cluster was acceptable. Hence, the resulting clusters were acceptable. Comparing the years 2015 and 2020 shows a worsening in the similarity of observations with other observations in the same cluster in most cases. However, this decrease was minimal (see Tables 6 and 7, Appendix 2).

In short, based on the selected facilitators, the cluster analysis revealed four groups of countries. The question is then, what characteristics were used to create these groups? Moreover, were there changes in the clusters from 2015 to 2020? The following section answers these questions. It also suggests possible causes of these movements.

### Evolution of SDGs and innovation

Changes were observed in the clusters for 2015 and 2020. For 2015, Clusters 1, 2, 3, and 4 consist of 27, 47, 24, and 24 countries, respectively. Cluster 1 has 18 African countries (e.g., Ethiopia, Guinea, and Senegal), two American countries (Guatemala and Honduras), and seven Asian countries (most notably India, Nepal, and Pakistan). This cluster is mainly formed of African countries. These countries show the highest values in the GINI index, indicating that the income inequality of these countries is higher. Cluster 2 is composed of 11 countries from Europe (e.g., Armenia, Montenegro, and North Macedonia), 12 Latin American countries (most notably Argentina, Brazil, and Paraguay), 16 Asian countries (e.g., China, Lebanon, and Vietnam), and eight African countries (most notably Algeria, Egypt, and Morocco). Cluster 2 is more varied than Cluster 1 because of the greater number of countries from different continents. On average, this group of countries presents a better GINI index performance, reflecting a better income distribution compared to the countries in Cluster 1.

**Fig. 3:**
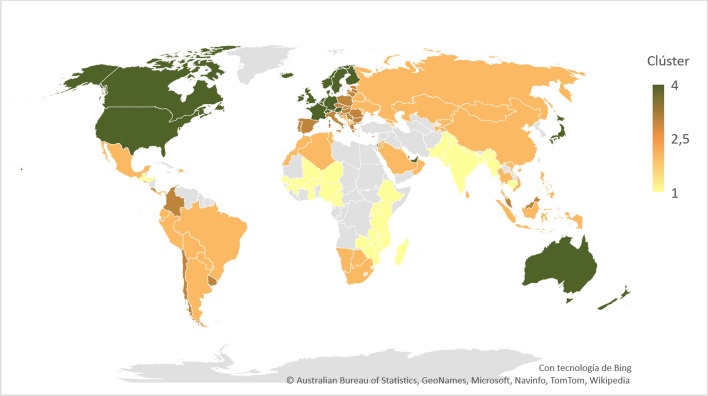
Composition of clusters for 2015

Cluster 3 has 18 European countries (e.g., Poland, Italy, and Spain), four Latin American countries (e.g., Chile and Colombia), one Asian country (Malaysia), and one African country (Mauritius). Finally, Cluster 4 has 14 European economies (e.g., Austria, France, and Switzerland), two American countries (Canada and the USA), six Asian countries (e.g., Israel, Japan, and United Arab Emirates), and two countries from Oceania (Australia and New Zealand). Although both clusters are predominantly European, they differ. Cluster 3 primarily consists of Eastern and Southern European countries, which tend to have lower levels of wealth than Central or Western European countries. These two clusters generally tend to have lower values in the GINI index (i.e., better performance). Income inequality levels are lower than in Clusters 1 and 2. Figure 3 illustrates the composition of each cluster for 2015.

**Fig. 4:**
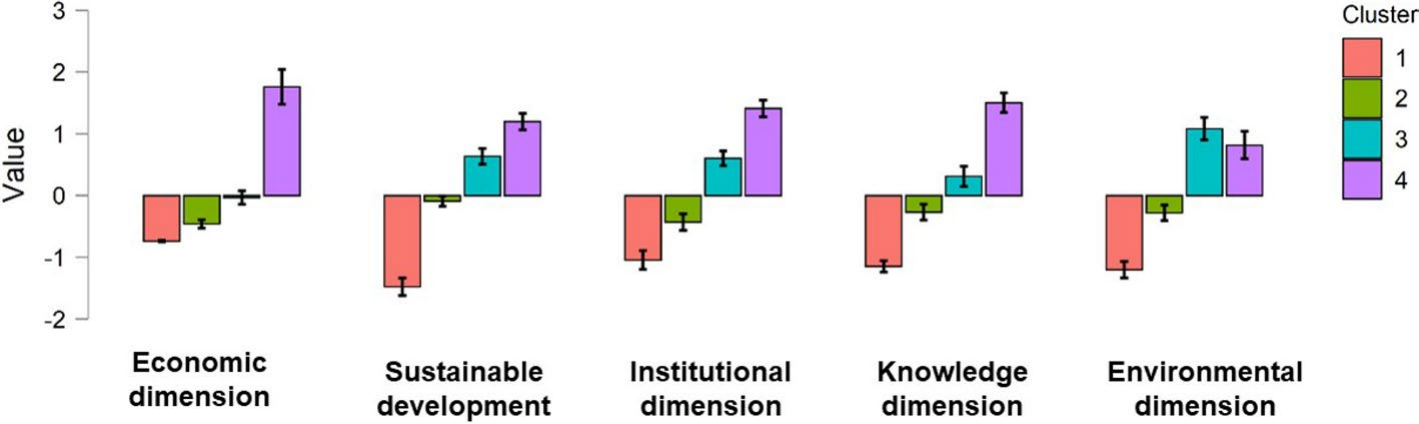
Cluster mean plot for 2015

Figure 4 shows the cluster mean for each predictor variable. It classifies the groups based on their innovation facilitators (dimensions) and degree of sustainable development attainment. Cluster 4 has high scores for these variables, particularly sustainable development and economic, institutional, and knowledge facilitators. Despite not having the highest score for environmental facilitators, the value is still high. Given the level of innovation facilitators and high SDG achievement of the countries in this group, Cluster 4 is labeled as “Very high innovative facilitators: very high sustainable development”. Cluster 3 has the second highest values in the sample, except for environmental facilitators. Therefore, this cluster is labeled as “High innovative facilitators: high sustainable development”. Cluster 1 has the lowest mean values for all predictor variables, denoting lower national development. Hence, this cluster is labeled as “Low innovative facilitators: low sustainable development”. Finally, Cluster 2 is defined as “Medium innovative facilitators: medium sustainable development” because its levels of sustainable development and innovation facilitators are located around zero.

Figure 4 shows the lower levels of environmental facilitators in Cluster 4 than in Cluster 3. Therefore, environmental standards are lower when income is higher, suggesting that economic growth destroys the environment and its ability to conserve and regenerate itself. This finding is consistent with the approach of post-growth authors (e.g., Daly, 1974, 2018; Kallis et al., 2018). Contrary to the post-growth view, the environmental Kuznets curve implicitly suggests that absolute decoupling is possible (Otero et al., 2020).

The results of the linear regression analyses by clusters and those shown in Fig. 4 suggest that the role of economic growth in sustainable development is limited. Economic growth is essential in the early stages of a country’s sustainable development. In the later stages, countries may experience a simultaneous improvement in economic and environmental conditions. Nevertheless, once a certain level of economic growth has been achieved, its progress generates environmental destruction and degradation.

**Fig. 5:**
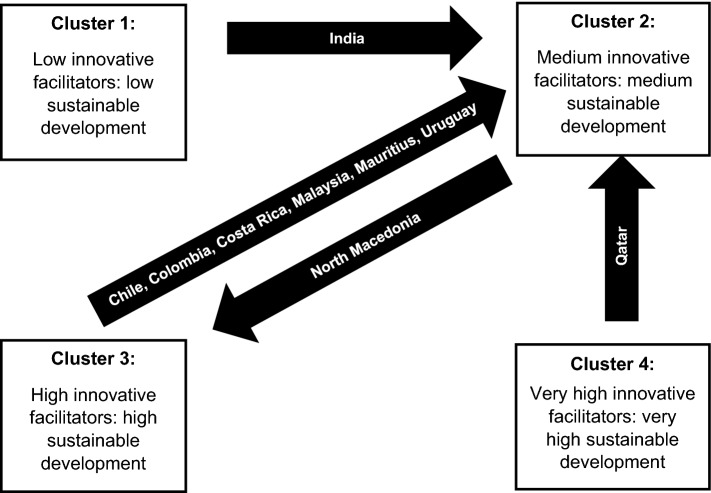
Country movements between clusters from 2015 to 2020

For 2020, Clusters 1, 2, 3, and 4 consist of 26, 54, 19, and 23 countries, respectively. India moved from the “Low innovative facilitators: low sustainable development” cluster (Cluster 1) to the “Medium innovative facilitators: medium sustainable development” cluster (Cluster 2). Chile, Colombia, Costa Rica, Malaysia, Mauritius, and Uruguay shifted from the “High development” cluster (Cluster 3) to Cluster 2, and Qatar moved from the “Very high innovative facilitators: very high sustainable development” cluster (Cluster 4) to Cluster 2. Finally, North Macedonia moved from the “Medium development” cluster to the “High development” cluster. Figure 5 illustrates these movements.

Given these results, the question is, what are the possible causes of the movements between clusters? The data indicate that India may have increased its sustainable development achievement because, although the level of its environmental facilitators declined, its level of wealth, institutional, and knowledge facilitators improved. Similarly, North Macedonia’s shift may be explained by the large improvement in economic, institutional, and environmental facilitators to offset the decrease in knowledge facilitators. In contrast, Qatar moved to the “Medium innovative sustainable development” cluster because it had lower values for wealth and institutional, knowledge, and environmental facilitators in 2020 than in 2015.

Although Chile, Colombia, Costa Rica, Malaysia, Mauritius, and Uruguay improved their SDG Index scores, they moved to the “Medium innovative facilitators: medium sustainable development” cluster. This finding highlights one of the limitations of this paper because not all possible variables affecting sustainable development were included in the model. Additionally, the movement of these regions may be explained by the change in the innovation facilitators of the “High innovative sustainable development” countries. Consequently, by 2020, countries in these clusters had distanced themselves from these six countries. In particular, while Colombia’s economic and institutional facilitators increased, its knowledge and environmental facilitators decreased. Similarly, Chile and Costa Rica increased their level of economic and knowledge facilitators, while the value of environmental and institutional facilitators decreased. Finally, Malaysia, Mauritius, and Uruguay saw only their value of environmental facilitators decrease.

## Conclusions

The aim of this study was to define clusters of countries according to their sustainable development and innovation facilitators for the years 2015 and 2020 and then examine the movements of countries between these clusters. Given the clear link between sustainable development and innovation facilitators and the objective of the paper, data from 122 countries were collected from the World Bank, GII, and SDG Index databases to identify countries’ innovation and sustainable development. Subsequently, cluster analysis was conducted to identify clusters according to countries’ sustainable development and innovation facilitators for the years 2015 and 2020 and to examine the movements of countries between these clusters. The results reveal four clusters consisting of countries with similar sustainable development and economic, institutional, knowledge, and environmental facilitators. Linear regression analysis shows that the variables in the model have the power to explain the level of sustainable development.

Each group has unique qualities. Clusters 1, 2, 3, and 4 are labeled as “Low”, “Medium”, “High”, and “Very high innovative facilitators and sustainable development,” respectively. Countries with a higher degree of achievement of the SDGs have high values for the independent variables related to institutional, knowledge, and environmental facilitators. In addition, there is a close link between the level of wealth, institutional, knowledge facilitators. These facilitators in turn have strong relationships with each other. These strong relationships may indicate that countries with higher economic levels invest more resources in institutional, educational, and research systems based on innovation to drive national development. These results suggest that richer economies allocate greater resources to promote innovation, making it a key element in driving sustainable development. Such a finding is in line with those of Husted (2005), who claimed that countries with stronger economies have more developed institutional and social capacities for sustainability because their strong economies provide more technology and resources for sustainable initiatives (Baughn et al., 2007; Reverte, 2022). Sustainable development means reorienting the progress of knowledge and technology, which should be neither eliminated nor interrupted (Schneider et al., 2010). Therefore, economic development, green technologies, and eco-innovations would have a positive relationship (Elgin et al., 2022).

Nevertheless, it is crucial to highlight the limited power of economic growth. This limitation is determined by the characteristics of the ecosystem and the biophysical world. The incompatibility between economic growth and biophysical limits leads to the loss of ecosystem value (Daly, 2018), reducing efficiency (Daly, 1974). Economic growth also accelerates biodiversity loss, climate change, and excessive waste and resource extraction (Kallis et al., 2012; Martínez-Alier et al., 2010). Any growth that attempts to exceed these limits generates environmental destruction and degradation (Fournier, 2008). Therefore, ecological sustainability declines, hindering the achievement of sustainable development. These conclusions indicate that the view of sustainable development adopted and promoted by international institutions is incomplete, which would fit with the perspective of Purvis et al. (2019). While sustainable development could comprise the three dimensions of sustainability (economic, social, and environmental), the economic dimension should be controlled according to environmental and social constraints. Therefore, society’s ability to meet its basic needs and live in harmony with nature and the environment could be preserved. The excessive use of energy, materials, and resources encouraged by mass consumption and production could thus be avoided.

Nine countries moved between clusters, namely India, Chile, Colombia, Costa Rica, Malaysia, Mauritius, Uruguay, North Macedonia, and Qatar. These movements were due to changes in the innovation facilitators for 2020 that increased inequalities between countries and affected the clusters with respect to those for 2015. The movements were also due to possible changes in the innovation facilitators of most of the countries in the cluster for 2015, which also increased inequalities.

In short, the achievement of the SDGs seems to depend on the level of wealth of countries, with the most developed economies showing the greatest capacity for innovation. In turn, innovation is closely related to sustainable development. The results underscore the idea that implementing sustainability in countries is not a low-cost strategy. However, the poorest and neediest regions cannot be left behind. The adoption of global measures and collaboration between more developed economies to favor innovation in developing economies could reduce inequalities between countries and boost the development of poorer regions that are unable to meet basic needs. Through stronger and more effective innovation facilitators, poorer regions could narrow the lag in technology and knowledge and thus close the sustainable development gap (Raffer & Singer, 2002). Nevertheless, when implementing these new sustainable development strategies and instruments, it is essential to consider the finite nature of economic growth and the limits of the physical ecosystem. If a maximum level of economic growth is not established, it may be impossible to achieve sustainable development because unconstrained economic growth would lead to environmental degradation. This argument contradicts the environmental Kuznets curve, which suggests that the negative effects of economic growth on biodiversity increase only up to a point, after which they decrease. This decline occurs because high economic growth raises concern for the conservation and protection of biodiversity (Dietz & Adger, 2003; Otero et al., 2020).

### Implications

This paper has crucial theoretical and practical implications for scholars and policymakers. The grouping of countries into clusters shows that national innovation facilitators lead to different levels of innovation performance. These different innovation performance levels then influence sustainable development to a varying degree. Despite this grouping and the similarities among countries in the same cluster, national characteristics still differ. The context, circumstances, and situation of each country should be considered when designing innovation policies to foster sustainable development. Policymakers or scholars can analyze the evolution of sustainable development performance or movement among groups of countries according to the level of sustainable development to modify or adopt innovation policies and initiatives that promote sustainability. In these cases, where innovation constitutes a driving force, it is important to study innovation systems because each country or region possesses unique characteristics that shape policymaking. National or regional innovation systems involve a set of connections and relationships among different agents of the innovation process within national or regional boundaries (Cooke et al., 1997; Freeman, 1987). Therefore, although collaboration could foster innovation and sustainable development (Milana & Ulrich, 2022; Ukko et al., 2019), individual national characteristics should be considered because no policy model can be applied uniformly to all countries (Tödtling & Trippl, 2005). In addition, when implementing these innovation-based sustainable development policies, a country’s level of economic development must be monitored. Doing so prevents excessive economic growth from exceeding the limits of the ecosystem and, hence, environmental degradation and destruction. In short, three valuable insights can be gained from the conclusions of the paper: (1) the link between innovation and sustainable development, (2) the need to study the characteristics of each innovation system to apply sustainable development policies and initiatives tailored to each country or region and thus ensure the effectiveness and success of sustainability, and (3) the limited power of economic growth in the context of sustainable development.

### Limitations

This research is not exempt from limitations. First, the analysis was conducted for the years 2015 and 2020. However, as mentioned earlier, data on the SDG Index for 2015 were not available. Similarly, GDP per capita data were not available for the year 2020 because it is too recent. Also, the cluster analysis only included a small number of innovation facilitators. Others, such as technological, political, and market facilitators, may also influence the achievement of sustainable development. Aspects such as political and economic stability, the investment or financing context, and competitiveness (Morkovkin et al., 2019) could also affect sustainable development.

### Future research possibilities

Given these limitations, cluster analysis including 2020 GDP per capita data and other factors affecting countries’ sustainable development capacity should be performed to examine cluster creation and subsequent movements between clusters. Possible causes and consequences could thus be identified. Similarly, given the number and variety of SDGs, similar analysis could be performed by breaking the index down to focus on a specific SDG such as poverty. This analysis would provide crucial knowledge and insights to identify the innovation facilitators that encourage the achievement of sustainable development.

## Appendix

### Appendix 1: Multiple linear regression analysis

See Tables 4, 5 and 6.

**Table 4:** Coefficients of the MLR models for 2015

Coefficients 2015								
							Collinearity statistics	
Model		Unstandardized	Standard error	Standardized	*t*	*p*	Tolerance	VIF
1	(Intercept)	61.509	1.14		53.958	< .001		
2	(Intercept)	37.11	1.355		27.384	< .001		
	V2015 Human capital and research	0.752	0.038	0.874	19.739	< .001	1	1
3	(Intercept)	29.347	1.577		18.606	< .001		
	V2015 Human capital and research	0.546	0.043	0.635	12.634	< .001	0.55	1.818
	V2015 Ecological sustainability	0.366	0.051	0.358	7.118	< .001	0.55	1.818
4	(Intercept)	26.42	2.014		13.117	< .001		
	V2015 Human capital and research	0.469	0.054	0.545	8.65	< .001	0.337	2.964
	V2015 Ecological sustainability	0.321	0.054	0.314	5.921	< .001	0.478	2.094
	V2015 Institutional framework	0.113	0.05	0.149	2.277	0.025	0.315	3.178

The following covariate was considered but not included: V2015 GDP per capita (current US$); VIF = variance inflation factor

**Table 5:** Coefficients of the MLR models for 2020

Coefficients 2020								
							Collinearity statistics	
Model		Unstandardized	Standard Error	Standardized	*t*	*p*	Tolerance	VIF
1	(Intercept)	69.791	0.781		89.363	< .001		
2	(Intercept)	55.32	1.094		50.588	< .001		
	V2020 Human capital and research	0.446	0.03	0.801	14.653	< .001	1	1
3	(Intercept)	51.668	1.106		46.696	< .001		
	V2020 Human capital and research	0.312	0.034	0.56	9.254	< .001	0.612	1.633
	V2020 Ecological sustainability	0.245	0.038	0.387	6.391	< .001	0.612	1.633
4	(Intercept)	50.509	1.221		41.354	< .001		
	V2020 Human capital and research	0.367	0.042	0.658	8.699	< .001	0.381	2.626
	V2020 Ecological sustainability	0.259	0.038	0.408	6.742	< .001	0.596	1.679
	V2019 GDP per capita (current US$)	− 5.872e−5	2.785e−5	−0.149	−2.108	0.037	0.434	2.302

The following covariate was considered but not included: V2020 Institutional framework

*VIF* variance inflation factor

**Table 6:** ANOVA 2015 and 2020

Model		Sum of squares	df	Mean square	*F*	*p*
*2015*						
2	Regression	14,665.950	1	14,665.950	389.642	< .001
	Residual	4516.741	120	37.640		
	Total	19,182.691	121			
3	Regression	16,014.796	2	8007.398	300.793	< .001
	Residual	3167.895	119	26.621		
	Total	19,182.691	121			
4	Regression	16,148.103	3	5382.701	209.306	< .001
	Residual	3034.588	118	25.717		
	Total	19,182.691	121			
*2020*						
2	Regression	5775.957	1	5775.957	214.715	< .001
	Residual	3228.067	120	26.901		
	Total	9004.023	121			
3	Regression	6600.881	2	3300.440	163.433	< .001
	Residual	2403.143	119	20.194		
	Total	9004.023	121			
4	Regression	6688.122	3	2229.374	113.591	< .001
	Residual	2315.902	118	19.626		
	Total	9004.023	121			

The intercept model is omitted because no meaningful information can be shown

### Appendix 2: Cluster analysis

See Tables 7 and 8.

**Table 7:** 2015 Cluster details

Cluster	1	2	3	4
Size	27	47	24	24
Explained proportion within-cluster heterogeneity	0.14	0.39	0.17	0.31
Within sum of squares	17.74	48.61	21.09	38.62
Silhouette score	0.51	0.29	0.37	0.38
Centroid V2015 GDP per capita (current US$)	− 0.74	− 0.46	− 0.03	1.76
Centroid V2016 SDG Index score	− 1.48	− 0.09	0.637	1.20
Centroid V2015 Institutional framework	− 1.04	− 0.43	0.61	1.41
Centroid V2015 Human capital and research	− 1.15	− 0.27	0.31	1.50
Centroid V2015 Ecological sustainability	− 1.20	− 0.28	1.08	0.82

The between sum of squares of the four-cluster model is 478.93; the total sum of squares of the four-cluster model is 605

**Table 8:** 2020 Cluster data

Cluster	1	2	3	4
Size	26	54	19	23
Explained proportion within-cluster heterogeneity	0.15	0.48	0.09	0.27
Within sum of squares	20.58	64.94	12.43	36.63
Silhouette score	0.50	0.28	0.51	0.36
Centroid V2019 GDP per capita (current US$)	− 0.75	− 0.41	0.09	1.74
Centroid V2020 SDG Index score	− 1.49	0.001	0.81	1.01
Centroid V2020 Institutional framework	− 1.04	− 0.35	0.63	1.48
Centroid V2020 Human capital and research	− 1.24	− 0.19	0.41	1.52
Centroid V2020 Ecological sustainability	− 1.04	− 0.36	1.49	0.79

The between sum of squares of the four-cluster model is 470.42; the total sum of squares of the four-cluster model is 605

## Declarations

### Data availability

The data sets that provided the results presented in this paper are available from the corresponding author on reasonable request.
